# Applying Deep Transfer Learning to Assess the Impact of Imaging Modalities on Colon Cancer Detection

**DOI:** 10.3390/diagnostics13101721

**Published:** 2023-05-12

**Authors:** Wael Alhazmi, Turki Turki

**Affiliations:** Department of Computer Science, King Abdulaziz University, Jeddah 21589, Saudi Arabia

**Keywords:** deep learning, transfer learning, classification, colon cancer, medical imaging

## Abstract

The use of medical images for colon cancer detection is considered an important problem. As the performance of data-driven methods relies heavily on the images generated by a medical method, there is a need to inform research organizations about the effective imaging modalities, when coupled with deep learning (DL), for detecting colon cancer. Unlike previous studies, this study aims to comprehensively report the performance behavior for detecting colon cancer using various imaging modalities coupled with different DL models in the transfer learning (TL) setting to report the best overall imaging modality and DL model for detecting colon cancer. Therefore, we utilized three imaging modalities, namely computed tomography, colonoscopy, and histology, using five DL architectures, including VGG16, VGG19, ResNet152V2, MobileNetV2, and DenseNet201. Next, we assessed the DL models on the NVIDIA GeForce RTX 3080 Laptop GPU (16GB GDDR6 VRAM) using 5400 processed images divided equally between normal colons and colons with cancer for each of the imaging modalities used. Comparing the imaging modalities when applied to the five DL models presented in this study and twenty-six ensemble DL models, the experimental results show that the colonoscopy imaging modality, when coupled with the DenseNet201 model under the TL setting, outperforms all the other models by generating the highest average performance result of 99.1% (99.1%, 99.8%, and 99.1%) based on the accuracy results (AUC, precision, and F1, respectively).

## 1. Introduction

**Figure 1:**
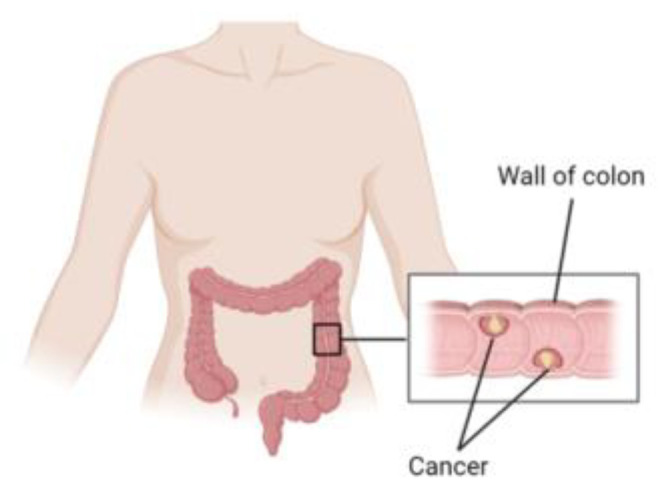
Colon cancer in the wall of the colon. Figure created with Biorender.com (accessed on 1 March 2023).

Cancer is an oftentimes rapidly spreading disease that drastically affects human health [1]. One of the most common types of cancer is colon cancer, which is sometimes caused by polyps in the colon wall, as shown in Figure 1 [2]. Colon cancer is the second and third most prevalent cancer in terms of death and incidence rates, respectively [3]. Consequently, previous studies have proposed many methods for improving the detection of colon cancer [4,5,6,7]. Medical imaging is one method used for detecting colon cancer. However, dealing with large numbers of medical images causes difficulties for specialists, which results in delays in the detection of colon cancer and, thus, delays in treatment. Therefore, automating the detection of colon cancer using deep learning (DL) attends to these challenges effectively.

Patino-Barrientos et al. [8] employed the VGG16 DL model to classify colon polyps as either malignant or nonmalignant, using an image dataset that consisted of 600 colonoscopy-derived images (from a private institution) based on Kudo’s method. The VGG16 model was utilized in two different ways, as demonstrated as follows: for the first situation, a pre-trained VGG16 model on ImageNet was used for the feature extraction, freezing layers pertaining to the convolutional feature extraction while changing the densely connected classifier to address the new binary classification problem. The convolutional feature extraction of the VGG16 model was then applied to the training data related to colon cancer, and the resulting features were used as inputs for the densely connected classifier to induce the model to perform predictions of unseen colon cancer images. For the second situation, a pre-trained VGG16 with fine tuning was used, which freezes the bottom layers while unfreezing the remaining layers. Compared to other machine-learning-based methods that use hand-crafted features of histograms of an oriented gradient, the results from a testing subset of the dataset demonstrate the superiority of the VGG16 model. Sarwinda et al. [9] aimed to classify colon cancer as malignant or benign using histology-based images. They utilized ResNet-based DL models, namely ResNet-18 and ResNet-50. Their approach worked as follows: firstly, the images were pre-processed using the contrast limited adaptive histogram equalization technique to generate improved images. Then, employing ResNet-18 and ResNet-50 and using feature extraction, they froze all the layers except for the densely connected classifier to deal with the binary classification problem. Features were extracted from pre-processed training images and given to the densely connected classifier, which was followed by the performing of predictions on the testing images. In terms of the evaluation, the dataset was divided into training and testing three times according to user-specified percentages. The reported results demonstrated the feasibility of ResNet-based DL models.

Ponzio et al. [10] aimed to classify colon cancer based on histological images. They utilized the VGG16 DL model in three different ways, including transfer learning. The first suggested model (a fully trained VGG16) consisted of training a VGG16 from scratch on colon cancer data and performing predictions of unseen histology-based images that were related to colon cancer. The second suggested model (a pre-trained VGG16 model on ImageNet with feature extraction) was applied to histology-based training images to extract features, and provided with corresponding labels for the machine learning algorithm (SVM). The induced SVM model was then applied to a testing set (consisting of feature vectors constructed from a pre-trained VGG16) to generate predictions. The third suggested model (a pre-trained VGG16 model on ImageNet with fine tuning) froze some layers while unfreezing the remaining layers. The experimental results demonstrated that pre-trained VGG16 models utilizing transfer learning (i.e., the second and third VGG16 models) outperformed the supervised learning approach of the VGG16 which was fully trained from scratch. Basha et al. [11] developed a CNN called RCCNet to classify colon cancer nuclei into four categories: miscellaneous, fibroblast, epithelial, and inflammatory. Their developed model was compared with various DL models: WRN, GoogLeNet, AlexNet, softmaxCNN, and softmaxCNN_IN27, and their proposed model achieved the best performance results. Ribeiro et al. [12] used CNN with data augmentation to classify colon images into two classes: healthy and abnormal. The experimental results demonstrated the good performance of the utilized CNN.

The problem with detecting colon cancer using medical images depends on the data-driven methods used and the images generated by an imaging modality. Unlike previous studies that have focused on evaluating the performance behavior of DL models in terms of detecting colon cancer [8,9,10,11,12], our contributions can be summarized as follows:

(1) We utilized three imaging modalities, namely, CT [13], colonoscopy [14], and histology [15,16], with five DL architectures, including VGG16 [17], VGG19 [17], ResNet152V2 [18], MobileNetV2 [19], and DenseNet201 [20].
(2) We comprehensively reported the performance behavior for the detection of colon cancer, including generated images via different modalities coupled with DL models in the transfer learning setting. Moreover, we constructed 26 ensemble DL models and compared their performance against the 5 studied DL models.
(3) We identified the best overall imaging modality and DL model for the detection of colon cancer. Specifically, our results reported that colonoscopy-based images outperformed CT-based (and histology-based) images when coupled with DL models.
(4) Our reported results demonstrate the superiority of DenseNet201 compared to 30 other DL models, including 4 DL methods and 26 ensemble DL models. According to the average performance results, measured using a 5-fold cross-validation of the whole dataset of colonoscopy-based colon cancer images, DenseNet201 generated the highest average accuracy of 99.1%, the highest average area under the ROC curve of 99.1%, the highest average F1 of 99.1%, and the highest average precision of 99.8%. Since the 26 ensemble DL models generated inferior performance results, we moved their results into the Supplementary Materials File.

## 2. Materials and Methods

### 2.1. Datasets

This study used four publicly available datasets for detecting colon cancer. Firstly, we used the Cancer Genome Atlas Colon Adenocarcinoma Collection (TCGA-COAD) dataset of CT imaging modalities (accessible at https://doi.org/10.7937/K9/TCIA.2016.HJJHBOXZ accessed on 6 January 2023), which includes 8387 CT images of colon cancer [21,22,23]. Secondly, we used the CT COLONOGRAPHY dataset of CT imaging modalities (accessible at https://doi.org/10.7937/K9/TCIA.2015.NWTESAY1 accessed on 6 January 2023), which includes 941,771 CT images, 268,652 of which are relevant to the current field of study [24,25,26]. Thirdly, we used the HyperKvasir Dataset of colonoscopy imaging modalities (accessible at https://doi.org/10.17605/OSF.IO/MH9SJ accessed on 6 January 2023), which includes 10,662 images and 374 videos that represent 23 and 30 categories, respectively, and 99,417 undefined images. Among the identified dataset, there are four videos of an instance of colon cancer and one video of a normal colon [27,28]. Fourthly, we used the NCT-CRC-HE-100K-NONORM Dataset of histology imaging modalities (accessible at https://search.datacite.org/works/10.5281/zenodo.1214456 accessed on 6 January 2023), which includes 100,000 histology images and 23,080 images related to our study, which were divided into 14,317 images of instances of colon cancer and 8763 images of normal colons [29].

### 2.2. Pre-Processing

**Figure 2:**
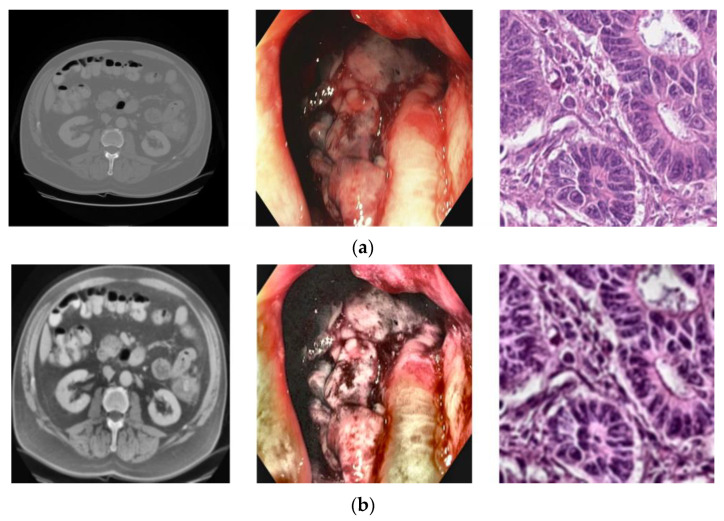
From left to right, the colon images of CT, colonoscopy, and histology, respectively, (**a**) before and (**b**) after pre-processing.

Pre-processing is a necessary phase of a medical image. It significantly affects the prediction results for colon cancer [30]. The datasets were obtained from various sources and techniques, including a subset of videos and poor-quality images with highlighted information, black borders, blurred contrast, and noise, which could influence the learning and prediction of the model. Therefore, we applied pre-processing to clean datasets, enhanced medical image conversion, generated a dataset of images from the videos, deleted blurred colon images, improved image quality, removed unwanted objects, and balanced class distribution. Firstly, we cleaned the datasets of lesions that were unrelated to our study. Then, we generated an image dataset for the colonoscopy technique by extracting frames from videos of the HyperKvasir dataset depending on FPS [31]. Thereafter, we removed the highlighted information by converting the color colon images to grayscale and using the THRESH_BINARY method to generate a binary mask and distinguish high and low pixel values; this was followed by the inpainting technique, which reconstructs the colon image using nearby pixels [32,33]. Next, we processed highly unbalanced datasets using a random undersampling technique that randomly selects samples from the majority class to equate to the minority classes [34]. Table 1 shows the number of images used for detecting colon cancer after applying the random undersampling method. Additionally, we enhanced the contrast of images using the CLAHE method followed by the Gaussian blur technique to remove any noise that the CLAHE method may have caused [35,36,37]. Moreover, we removed the black borders from the images to focus on processing the important features [38]. Finally, we changed the multiscale of images to fit the inputs of the CNN models using the INTER_LINEAR technique to 224 × 224 [39]. Figure 2 shows images of the colon before and after the pre-processing procedure.

### 2.3. Deep Learning Approach

**Figure 3:**
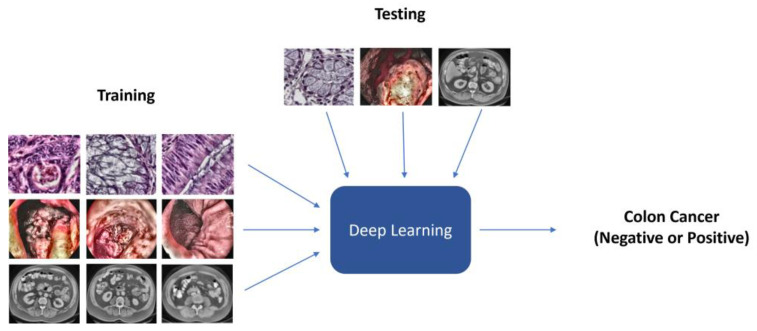
An illustration of the DL approach used for the discrimination between normal colon (negative) and colon cancer (positive) to detect colon cancer.

**Figure 4:**
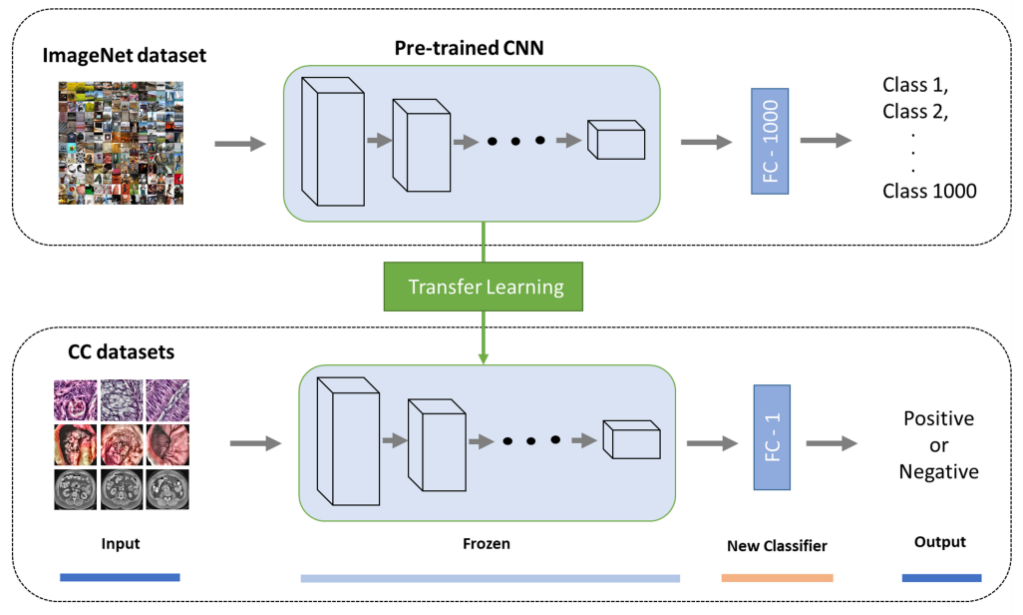
Transfer learning using feature extraction technique in developing the proposed CNN models.

The DL approach used for predicting colon cancer and distinguishing between normal colons (negative) and colon cancer (positive) is shown in Figure 3. $S=\left\{\left(x_{i},y_{i}\right)\right\}\begin{array}{c} m\\ i=1 \end{array}$ is a training set that includes *m*-labeled images obtained from various imaging modalities. Each training example has a class label (0 or 1), where 0 indicates a normal colon and 1 indicates colon cancer. This study used five pre-trained CNN models, including VGG16, VGG19, ResNet152V2, MobileNetV2, and DenseNet201. We adapted the five DL models to our problem using a transfer learning method based on the ImageNet dataset and feature extraction technique [40], whereby all layers were frozen with weights of ImageNet except for the last layer, which was replaced by a new dense layer that had one neuron and sigmoid activation and was trained independently on each of the colon cancer datasets, as shown in Figure 4. Each of the five DL models were trained independently on processed images of a given modality. Then, the unseen datasets were tested on the trained models of the same modality to generate predictions mapped to 0 and 1 as follows: if the prediction is greater than 0.5, it is set to 1, which thus indicates colon cancer; otherwise, it indicates a normal colon. 

## 3. Results 

### 3.1. Classification Methodology

For each image dataset, we investigated the performance of three imaging modalities (CT, histology, and colonoscopy) through five DL models (VGG16, VGG19, ResNet152V2, MobileNetV2, and DenseNet201) for predicting colon cancer. The five DL models were utilized in the transfer learning setting to address the classification task. After training, the DL models were applied to the testing images to generate predictions, which were mapped according to the following specified thresholds: 0 (normal colon) or 1 (colon cancer). Furthermore, we constructed 26 ensemble DL models. Since the 26 ensemble DL models did not outperform DenseNet201, we recorded their results in the Supplementary Materials File. To evaluate the performance of the models, we used five performance metrics: accuracy (*ACC*), precision (*PRE*), recall (*REC*), *F*1, and area under the ROC curve (AUC) [40,41]. To validate the performance of the DL models over the entire dataset, we applied a five-fold cross-validation by partitioning each dataset into five folds. For each run, we assigned five folds: four for the training set and one for the test set, where the prediction was applied to the testing fold. Finally, we reported the average performance results of the five runs using the following performance metrics:

(1) $$\;\;\;\;\;\;ACC=\frac{TP+TN}{TP+TN+FP+FN}$$

(2) $$PRE=\frac{TP}{TP+FP}$$

(3) $$REC=\frac{TP}{TP+FN}$$

(4) $$\;\;\;\;\;\;\;F1=\frac{2*PRE*REC}{PRE+REC}$$

where *TP* stands for true positive, referring to the number of colon cancer images that were correctly classified as colon cancer. *FN* stands for false negative, referring to the number of colon cancer images that were incorrectly classified as a normal colon. *TN* stands for true negative, referring to the number of normal colon images that were correctly classified as a normal colon. *FP* stands for false positive, referring to the number of normal colon images that were incorrectly classified as colon cancer.

### 3.2. Implementation Details

In this experiment, we used the Spyder editor (Version 4.2.5), which we accessed using Anaconda (Version 4.12.0) in Python (Version 3.8.8) [42,43]. We used the Keras library to run five DL models [44]. The datasets were processed in the pre-processing stage using OpenCV and NumPy libraries [45,46]. The training and testing of the DL models were conducted on the NVIDIA GeForce RTX 3080 Laptop GPU with 16 GB GDDR6 VRAM. For assessing the five DL models, we used the Sklearn library [46]. To obtain the box plot statistics for the training and testing phases, we utilized ggplot2 in R [47]. 

### 3.3. Classification Results

The datasets used in this study included 5400 processed images that were divided equally between normal colon and colon cancer and related to three types of medical images. Based on that, we assessed the image datasets obtained from three imaging modalities using five DL models (and we moved twenty-six ensemble DL models to the Supplementary Materials File because they produced inferior results), which was then followed by reporting their performances using a five-fold cross-validation. 

#### 3.3.1. Training Results

**Figure 5:**
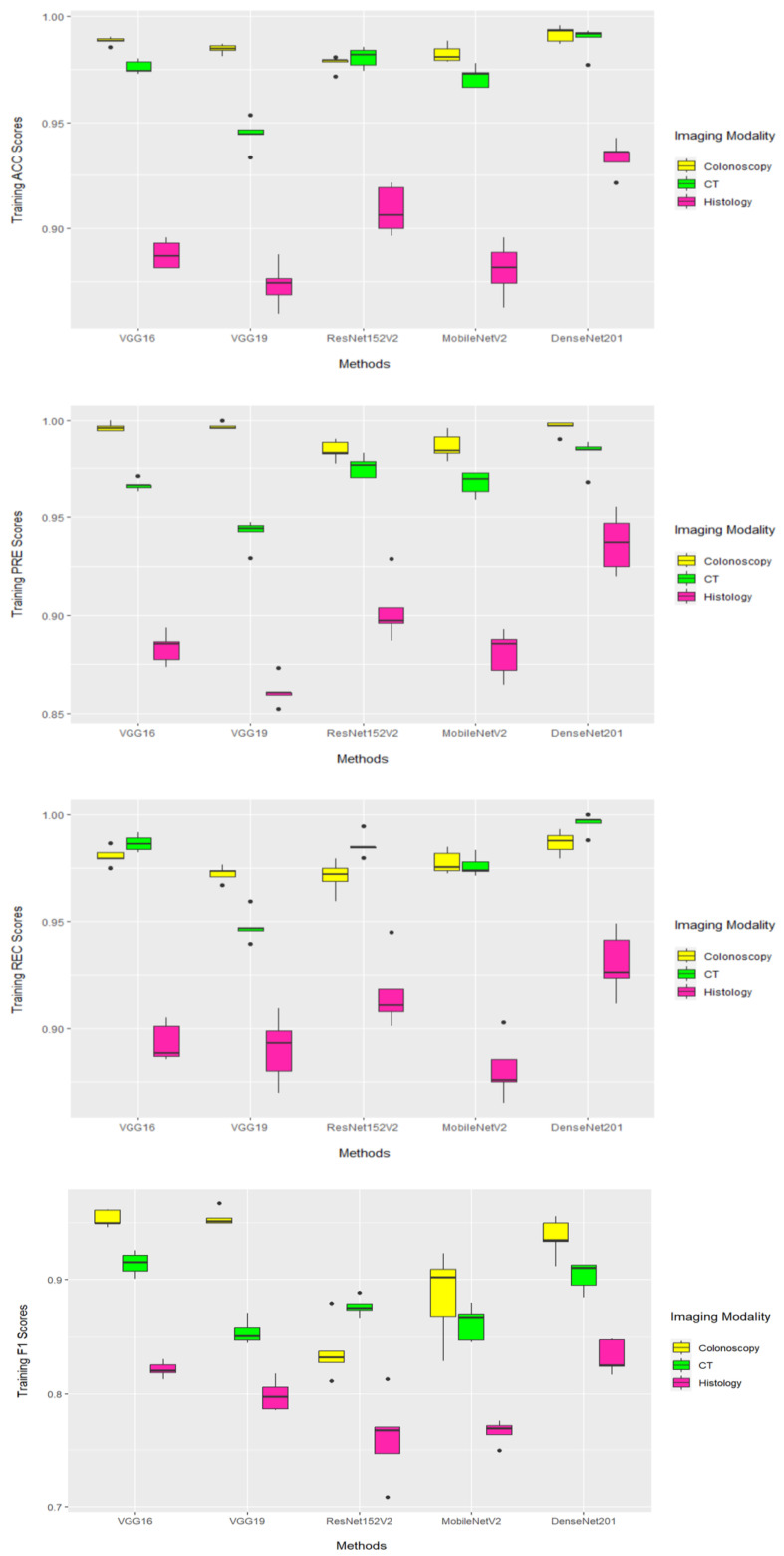
The training performance results based on 5-fold cross-validation using ACC, PRE, REC, and F1 performance measures. Boxplot showing the performance measures of five DL models for CT, histology, and colonoscopy imaging modalities.

Figure 5 illustrates the performance of the DL models when applied to images derived from imaging modalities on the training sets during a five-fold cross-validation based on the ACC, PRE, REC, and F1 performance measurements. The boxplots showed that DenseNet201 generated the highest performance results, according to ACC and PRE, when coupled with images derived from colonoscopy and CT imaging modalities. When DenseNet201 was coupled with images derived from CT imaging modality, it generated the highest results. The DL models achieved poor performance results when they were coupled with images that were derived from a histology imaging modality.

#### 3.3.2. Testing Results

**Figure 6:**
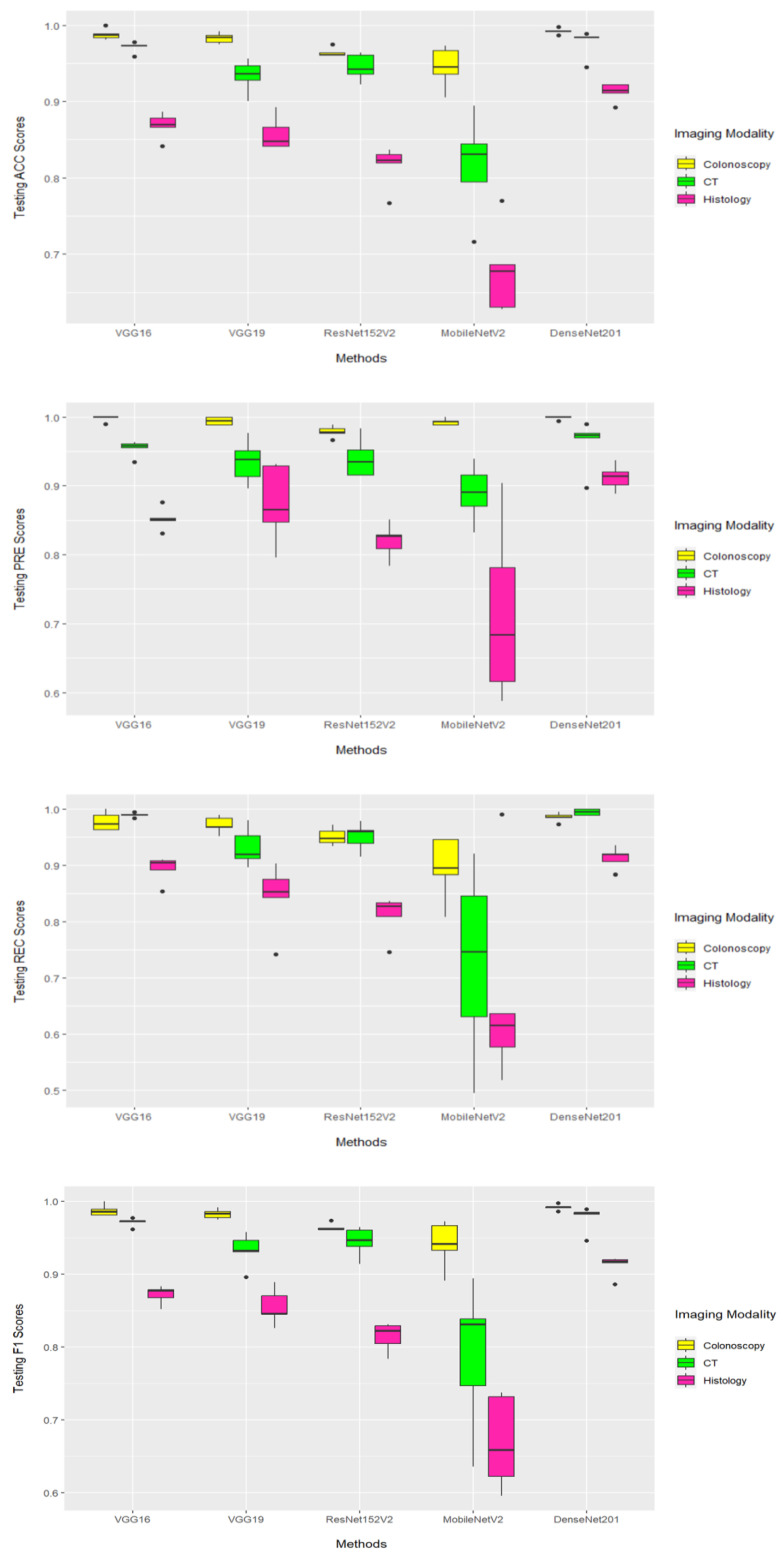
The test performance results based on 5-fold cross-validation using ACC, PRE, REC, and F1 performance measures. Boxplot showing the performance measures of five DL models for CT, histology, and colonoscopy imaging modalities.

**Figure 7:**
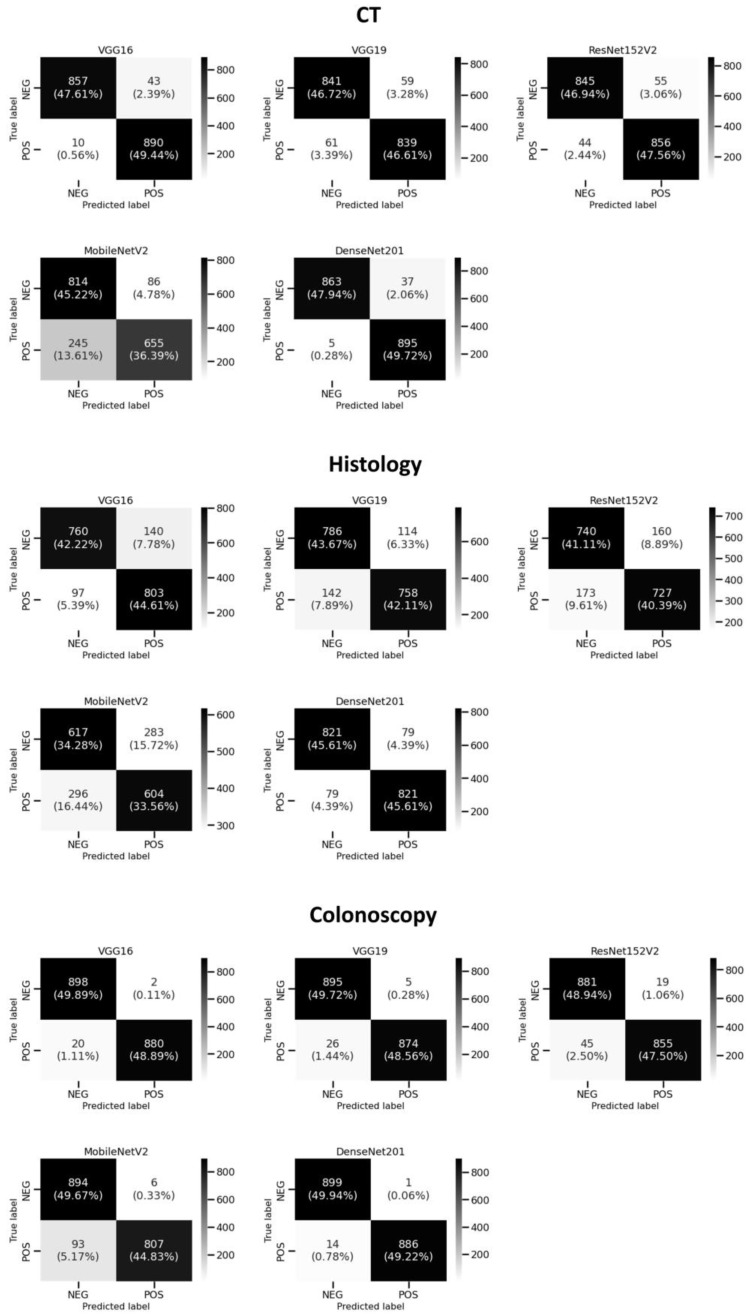
The combined confusion matrices of two categories (normal colon (NEG) and colon cancer (POS)) based on five DL models using five-fold cross-validation on the test sets for each of the CT, histology, and colonoscopy.

**Figure 8:**
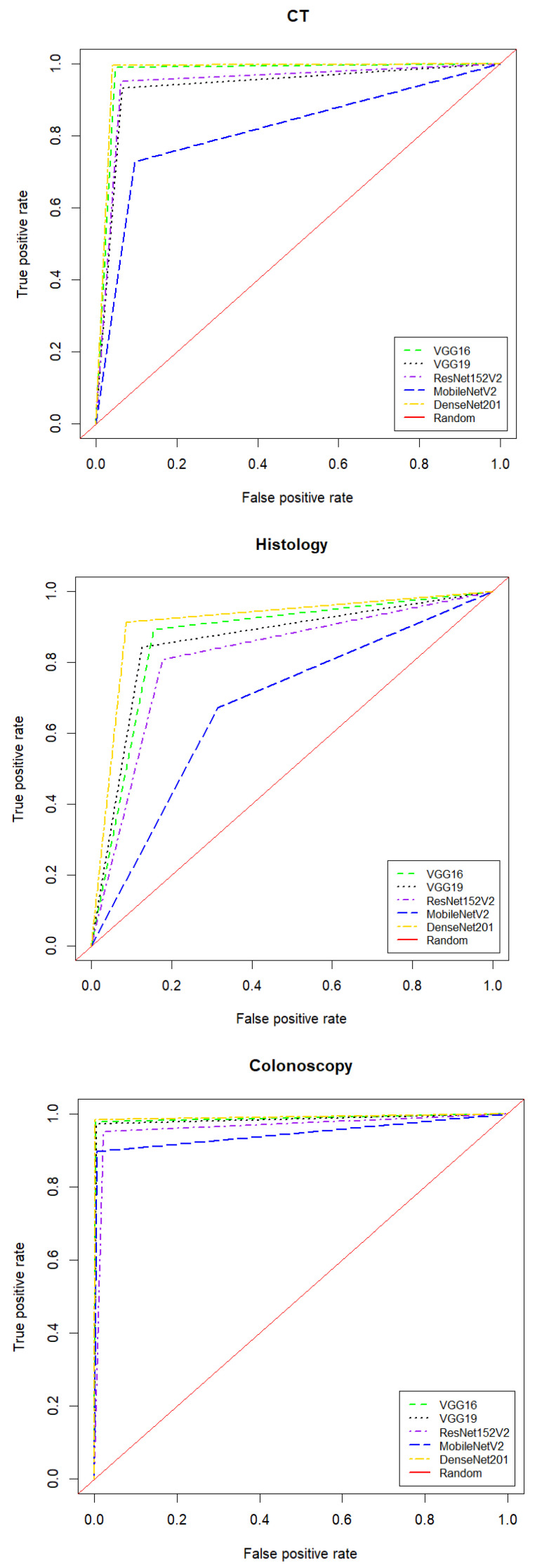
ROC curves for DL models applied to image datasets derived from the three studied imaging modalities using five-fold cross-validation. ROC: receiver operating characteristics.

Figure 6 shows that DenseNet201 achieved the best average performance results when coupled with images that were derived from colonoscopy and CT imaging modalities. Specifically, DenseNet201 (when coupled with colonoscopy-based images) achieved 99.1% (99.8% and 99.1%) according to ACC (PRE and F1, respectively). Moreover, it obtained the best average REC of 99.4% for images that were derived from a CT imaging modality, as shown in Table 2. For images derived from a histology imaging modality, MobileNetV2 achieved the lowest average performance results (66.6–71.4%) based on employed performance measures. According to Table 2, the colonoscopy imaging modality, when coupled with the DenseNet201 model, achieved the most reliable performance results. Figure 7 illustrates the combined confusion matrices of a five-fold cross-validation on the test sets. For each DL model and imaging modality, the sum of five test splits corresponds to the combined confusion matrices, and the sum of entries indicates that the whole dataset was used. Figure 8 displays the ROC curves for five DL models applied to the image datasets obtained from CT, histology, and colonoscopy imaging modalities. The DL model with the highest curve indicates the highest AUC results. It can be seen that DenseNet201 archives the highest AUC values, which are recorded in Table 2.

## 4. Discussion

Our DL system included four parts: (1) data acquisition; (2) data pre-processing; (3) the handling of the issue of binary classification under different medical imaging techniques, where we aimed to detect colon cancer by distinguishing between normal colon and colon cancer; and (4) the investigating of various imaging modalities through different DL models in the transfer learning setting. After the image dataset acquisition, which included 5400 images from normal colon and colon cancer of different imaging modalities, we provided the processed image datasets to DL models and reported the performance results using a five-fold cross-validation.

The technical contributions of this study are as follows: (1) the application of DL models to detect colon cancer under different imaging modalities; (2) the conducting of experimental studies in the transfer learning setting using processed datasets of 5400 images (900 of normal colons and 900 of colon cancer for computed tomography images; 900 of normal colons and 900 of colon cancer for histology images; and 900 of normal colons and 900 of colon cancer for standard colonoscopy images); (3) the inclusion of an extensive performance comparison of 5 DL models and 26 ensemble methods; and (4) the identification of the best DL model associated with images generated by an imaging modality. 

For an explanation pertaining to transfer learning, we passed the colon cancer image samples through the feature extraction part of a pre-trained CNN on ImageNet to extract the features, which were provided to a new densely connected classifier that was trained from scratch. In other words, we reused the feature extraction part of a pre-trained CNN on ImageNet by freezing the involved layers to extract the features from colon cancer images while changing the densely connected classifier of the pertained CNN on ImageNet to address the binary class classification problem in this study. It is worth noting that the term ‘feature extraction part’ refers to layers in the CNN that are related to feature extraction, such as convolutional and pooling layers. Additionally, freezing a layer prevents its weight from being updated [48]. It is evident that transfer learning is attributed to the weights kept in the feature extraction part of the pre-trained CNN.

In this study, we employed deep transfer learning models to (1) report the performance behavior of DL models when coupled with images generated via studied imaging modalities; (2) assess the feasibility of DL; and (3) promote the use of AI as a tool that can help doctors in the detection of colon cancer by identifying which imaging modality leads to high performance results when coupled with a DL model. All the studied datasets, which are cited in the datasets subsection, are labeled by domain experts and are publicly available. The colon cancer CT image dataset (and the other colon cancer datasets obtained from different modalities) consisted of 1800 images with a uniform class distribution. For the training phase during a 5-fold cross-validation, we utilized a batch size set to 20 as in [49,50], set the learning rate for the SGD optimizer to 0.0001 as in [51], and used binary_crossentropy as the loss function. Moreover, we trained the models for 20 epochs coinciding with Ref. [50]. We used the testing fold to assess the performance of each trained model. As the five-fold cross-validation ran five times, we reported the average performance on the testing folds. In other words, we utilized the five-fold cross-validation to report the performance on the whole dataset, as combining the images on the five testing folds corresponded to the 1800 images in the colon cancer CT image dataset. It is worth mentioning that during an iteration of a 5-fold cross-validation, the testing fold included 360 images from the 2 categories (180 images from each category), and the training splits included 1440 images from the 2 categories (720 images from each category).

For the ensemble methods, the 26 (i.e., $\left(\begin{array}{c} 5\\ 2 \end{array}\right)$ + $\left(\begin{array}{c} 5\\ 3 \end{array}\right)\;$+ $\left(\begin{array}{c} 5\\ 4 \end{array}\right)$ +$\;\left(\begin{array}{c} 5\\ 5 \end{array}\right)$) ensemble DL models were constructed using a majority vote as follows: for an ensemble of two DL models, where $\left(\begin{array}{c} 5\\ 2 \end{array}\right)$ was the number of different ensemble DL models, each ensemble consisted of two DL models out of five, and $\left(\begin{array}{c} n\\ r \end{array}\right)$ denoted the binomial theorem. The five DL models included VGG16, VGG19, ResNet152V2, MobileNetV2, and DenseNet201. Therefore, we created ten ensemble DL models by taking combinations of two out of the five DL models. For an ensemble composed of three DL models, $\left(\begin{array}{c} 5\\ 3 \end{array}\right)$ was the number of different ensemble methods, where each ensemble consisted of three DL models out of five. Therefore, we created ten ensemble DL models by taking combinations of three out of the five DL models. For the ensemble DL models composed of four DL models, $\left(\begin{array}{c} 5\\ 4 \end{array}\right)$ was the number of different ensemble DL models. Therefore, we created five ensemble DL models by taking combinations of four out of the five DL models. The last ensemble DL model consisted of five DL models. Therefore, we created one (i.e., $\left(\begin{array}{c} 5\\ 5 \end{array}\right)$) ensemble DL model. We used majority vote when making a prediction in each of the 26 ensemble methods. Since all the 26 ensemble DL models did not perform well compared to DenseNet201, we moved their results to the Supplementary Materials File.

## 5. Conclusions and Future Work

To assess image modalities for the task of colon cancer detection, we proposed using DL models under transfer learning. For the image dataset preparation, we performed the following tasks: cleaning, extracting frames, removing unwanted objects, handling imbalanced categories, image enhancement, noise removal, removing black borders, cropping, and resizing images. Then, several DL models (VGG16, VGG19, ResNet152V2, MobileNetV2, and DenseNet201) were coupled with colon cancer images from various imaging modalities (CT, histology, and colonoscopy) to discriminate between instances of normal colons and colon cancer. Each DL model was independently trained on the colon cancer image datasets of a given modality and then applied to the test set to perform predictions. For an assessment of the DL models, including the 26 ensemble-based DL models, we used a 5-fold cross-validation and several performance measures, including accuracy, precision, recall, and F1. Unlike histology-based (and CT-based) images, the experimental results demonstrated that DenseNet201 (under transfer learning with feature extraction) coupled with images derived from standard colonoscopy achieved the best average accuracy of 99.1%, the best average AUC of 99.1%, the best average precision of 99.8%, and the best average F1 of 99.1%.

Future work in this field should include the following: (1) the utilization of the presented deep transfer learning method to investigate other imaging modalities, such as MRI and PET, coupled with different pre-trained models, and (2) the expansion of the binary classification problem to attend to the multiclass classification problem in order to address classification tasks that are related to different cancer types.

## Figures and Tables

**Table 1:** The number of CT, histology, and colonoscopy images used for detecting colon cancer.

Dataset	Modality	Distribution	
		POS ^1^	NEG ^2^
TCGA-COAD	CT	900	-
CT COLONOGRAPHY	CT	-	900
NCT-CRC-HE-100KNONORM	Histology	900	900
HyperKvasir	Colonoscopy	900	900

^1^ POS refers to positive samples (cancer). ^2^ NEG refers to negative samples (normal).

**Table 2:** A performance comparison between the CT, histology, and colonoscopy imaging modalities using different deep learning (DL) models during the 5-fold cross-validation on test sets for accuracy (ACC), precision (PRE), recall (REC), F1, and area under the ROC curve (AUC). MACC is mean accuracy, MPRE is mean precision, and MREC is mean recall. MF1 is mean f1. MAUC is mean AUC. Bold represents the highest mean performance measure.

Imaging Modality	Method	MACC	MPRE	MREC	MF1	MAUC
CT	VGG16	0.970	0.954	0.988	0.971	0.970
	VGG19	0.933	0.935	0.931	0.933	0.933
	ResNet152V2	0.945	0.939	0.950	0.945	0.945
	MobileNetV2	0.816	0.889	0.727	0.798	0.816
	DenseNet201	0.976	0.961	**0.994**	0.977	0.976
Histology	VGG16	0.868	0.851	0.892	0.871	0.868
	VGG19	0.857	0.873	0.842	0.855	0.857
	ResNet152V2	0.815	0.819	0.810	0.813	0.815
	MobileNetV2	0.678	0.714	0.666	0.675	0.678
	DenseNet201	0.912	0.911	0.912	0.912	0.912
Colonoscopy	VGG16	0.987	0.997	0.977	0.987	0.987
	VGG19	0.982	0.994	0.971	0.982	0.982
	ResNet152V2	0.964	0.978	0.950	0.963	0.964
	MobileNetV2	0.945	0.992	0.895	0.942	0.945
	DenseNet201	**0.991**	**0.998**	0.984	**0.991**	**0.991**

## Data Availability

The publicly available datasets in this manuscript are as follows: The TCGA-COAD dataset is available at https://doi.org/10.7937/K9/TCIA.2016.HJJHBOXZ, accessed on 6 January 2023. The CT COLONOGRAPHY dataset is available at https://doi.org/10.7937/K9/TCIA.2015.NWTESAY1, accessed on 6 January 2023. The HyperKvasir dataset is available at https://doi.org/10.17605/OSF.IO/MH9SJ, accessed on 6 January 2023. The NCT-CRC-HE-100K-NONORM dataset is available at https://search.datacite.org/works/10.5281/zenodo.1214456, accessed on 6 January 2023.

## Supplementary Materials

The following supporting information can be downloaded at: https://www.mdpi.com/article/10.3390/diagnostics13101721/s1.

## Abbreviations

The following abbreviations are used in this manuscript:

DL	Deep Learning
CT	Computed Tomography
VGG	Visual Geometry Group
ResNet	Residual Neural Network
CNN	Convolutional Neural Network
SVM	Support Vector Machine
CLAHE	Contrast Limited Adaptive Histogram Equalization
Conv	Convolutional
FC	Fully Connected
CC	Colon Cancer
SGD	Stochastic Gradient Descent
MRI	Magnetic Resonance Imaging
PET	Positron Emission Tomography
ACC	Accuracy
PRE	Precision
REC	Recall
ROC	Receiver Operating Characteristic
AUC	Area Under the ROC Curve
